# Hydrogen sulfide in posthemorrhagic shock mesenteric lymph drainage alleviates kidney injury in rats

**DOI:** 10.1590/1414-431X20154057

**Published:** 2015-04-28

**Authors:** B. Han, Z.G. Zhao, L.M. Zhang, S.G. Li, C.Y. Niu

**Affiliations:** Institute of Microcirculation, Hebei North University, Hebei Zhangjiakou, China

**Keywords:** Hemorrhagic shock, Mesenteric lymph, Drainage, Kidney injury, Hydrogen sulfide, Inflammation

## Abstract

Posthemorrhagic shock mesenteric lymph (PHSML) is a key factor in multiple organ injury following hemorrhagic shock. We investigated the role of hydrogen sulfide (H_2_S) in PHSML drainage in alleviating acute kidney injury (AKI) by administering D,L-propargylglycine (PPG) and sodium hydrosulfide hydrate (NaHS) to 12 specific pathogen-free male Wistar rats with PHSML drainage. A hemorrhagic shock model was established in 4 experimental groups: shock, shock+drainage, shock+drainage+PPG (45 mg/kg, 0.5 h prehemorrhage), and shock+drainage+NaHS (28 µmol/kg, 0.5 h prehemorrhage). Fluid resuscitation was performed after 1 h of hypotension, and PHMSL was drained in the last three groups for 3 h after resuscitation. Renal function and histomorphology were assessed along with levels of H_2_S, cystathionine-γ-lyase (CSE), Toll-like receptor 4 (TLR4), interleukin (IL)-10, IL-12, and tumor necrosis factor (TNF)-α in renal tissue. Hemorrhagic shock induced AKI with increased urea and creatinine levels in plasma and higher H_2_S, CSE, TLR4, IL-10, IL-12, and TNF-α levels in renal tissue. PHSML drainage significantly reduced urea, creatinine, H_2_S, CSE, and TNF-α but not TLR4, IL-10, or IL-12. PPG decreased creatinine, H_2_S, IL-10, and TNF-α levels, but this effect was reversed by NaHS administration. In conclusion, PHSML drainage alleviated AKI following hemorrhagic shock by preventing increases in H_2_S and H_2_S-mediated inflammation.

## Introduction

Acute kidney injury (AKI) is a pathological process that commonly occurs in conditions such as hemorrhage, trauma, infection, or intoxication and contributes to the progression of internal milieu disorders, resulting in multiple organ failure (1-4). Recently, the relationship between lymph circulation and pathogenesis of serious diseases has received increasing attention, with evidence that under critical clinical conditions, the return of mesenteric lymph to the systemic circulation is a key factor in vital organ dysfunction and injury (5-7). The results obtained in our previous studies suggest that blocking the return of mesenteric lymph to the systemic circulation attenuates renal lesions induced by hemorrhage and lipopolysaccharide or severe hemorrhagic shock in a two-hit animal model (8-10). However, the underlying mechanisms remain to be discovered.

Hydrogen sulfide (H_2_S) is an endogenous gaseous signaling molecule involved in diverse biological processes such as inflammatory responses, energy metabolism, cell proliferation, apoptosis, and oxidative stress (11,12). Recent studies indicate that H_2_S is responsible for the inflammatory response and organ dysfunction following hemorrhagic shock (13). Pretreatment with D,L-propargylglycine (PPG), an inhibitor of cystathionine-γ-lyase (CSE), attenuates increases in plasma levels of tumor necrosis factor (TNF)-α, interleukin (IL)-6, and urea and reduces H_2_S concentration in the kidney following hemorrhagic shock (13). Francescato et al. (14) showed that treatment with PPG attenuates gentamicin-provoked effects such as macrophage infiltration in the renal cortex and interstitial lesions in renal tubules. These reports suggest that increased H_2_S levels that mediate AKI are induced by renal ischemia or nephrotoxic drugs. However, an H_2_S-induced inflammatory response related to AKI caused by the return of posthemorrhagic shock mesenteric lymph (PHSML) has not yet been reported.

In this study, PPG and the H_2_S donor sodium hydrosulfide hydrate (NaHS) were administered to rats subjected to hemorrhagic shock with PHSML drainage. The aim was to investigate the role of H_2_S in PHSML drainage in the protection of renal function. Changes in the levels of Toll-like receptor 4 (TLR4), IL-10, IL-12, and TNF-α are described, and the mechanism of PHSML drainage in AKI is discussed.

## Material and Methods

### Animals

Thirty healthy, specific pathogen-free male Wistar rats weighing 220-260 g were purchased from the Chinese Academy of Medical Sciences Animal Breeding Center (Beijing, China). The rats were housed in a climate-controlled facility with a 12-h light/dark cycle and free access to standard laboratory food and water for at least 1 week prior to the experimental procedures. The rats ware fasted and only allowed water for 12 h before the start of experimental procedures. Rats were randomly allocated to 5 groups: sham, shock, shock+drainage, shock+drainage+PPG, and shock+drainage+NaHS (n=6/group). All procedures involving animals were reviewed and approved by the Hebei North University Animal Care Committee and conformed to the guidelines of the National Institutes of Health. Maximum efforts were made to minimize animal suffering.

### Hemorrhagic shock model

Rats were anesthetized with pentobarbital sodium (1%, 50 mg/kg), and those in the shock+drainage+PPG and shock+drainage+NaHS groups were given PPG (45 mg/mL, 45 mg/kg; Sigma, USA) or NaHS (28 µmol/mL, 28 µmol/kg, Sigma) by intraperitoneal (*ip*) injection. Control animals (i.e., those in the sham, shock, and shock+drainage groups) were given *ip* injections of an equal volume of normal saline (1 mL/kg). The PPG and NaHS doses were the same as those used in previous studies (15,16). The right femoral vein was aseptically isolated, catheterized with polyethylene tubing containing heparin sodium (500 U/kg) for anticoagulation, and connected to an infusion pump (WZF-250F2, Zhejing University Medical Instrument Company, China) for fluid resuscitation. The bilateral femoral arteries were also isolated. A minimally heparinized polyethylene catheter was introduced into the left femoral artery to allow continuous monitoring of mean arterial pressure (MAP) using a biological signal acquisition system (RM6240BD, Chengdu Instrument, China). Another catheter was inserted into the right femoral artery for blood letting. All rats then underwent abdominal surgery to separate the mesenteric lymph duct from the surrounding connective tissues. Rats in the shock, shock+drainage, shock+drainage+PPG, and shock+drainage+NaHS groups were allowed to stabilize for 30 minutes and were then hemorrhaged rapidly from the right femoral artery with an automatic withdrawal-infusion machine (NE-1000; New Era Pump Systems Inc., USA) so that the MAP dropped to 40 mmHg within 10 min. MAP was maintained at this level for 60 min through the withdrawal or reperfusion of lost blood as required. The collected blood plus an equal volume of Ringer's solution were then reperfused within 30 min through the right femoral vein. After completion of resuscitation, the mesenteric lymph duct was cannulated, and PHSML was drained for up to 3 h in the shock+drainage, shock+drainage+PPG, and shock+drainage+NaHS groups. Rats in the sham group were anesthetized and cannulated as described above, but hemorrhage and resuscitation were not performed. At 3 h after resuscitation, a 3-mL blood sample was withdrawn from the abdominal aorta, and renal tissues were harvested for subsequent examination.

### Examination of renal function

Plasma was collected by centrifugation at 850 *g* for 10 min and stored at -75° to -80°C in a refrigerator (Thermo Electron, USA). Plasma urea and creatinine (Cre) levels were measured by an automatic biochemical analyzer (7600-110, Hitachi, Japan).

### Morphological observation of kidney

Renal tissues were fixed in 4% paraformaldehyde after harvesting from a specified location in the rat kidneys. Following dehydration in a graded alcohol series and paraffin embedding, the renal tissues were sectioned at 5 μm and stained with hematoxylin and eosin. Tissue morphology was examined by light microscopy (90i; Nikon, Japan) in 10 randomly chosen fields per sample and then photographed and analyzed using an image collection and analysis system (Eclipse, Nikon).

### Preparation of renal homogenate

The isolated renal tissues were homogenized in 1:9 (w/v) physiological saline for 30 s using an FJ-200 tissue homogenizer (Shanghai Specimen and Model Factory, China). The homogenates were centrifuged at 850 *g* at 0-4°C for 10 min (Labofuge 400R; Thermo Fisher Scientific, USA), and the supernatants were kept frozen at -75° to -80°C until they were used in subsequent assays.

### H_2_S assays of renal homogenates

A standard calibration curve was plotted using solutions containing different, known concentrations of NaHS. A linear regression equation (*y*=1532.5*x*+6.786, *r* ^2^=0.995) was derived to assay H_2_S in the renal homogenates as previously described (13). Briefly, 0.1 mL renal homogenate was added to a test tube containing 0.5 mL 10 g/L zinc acetate. After mixing, 0.5 mL p-phenylenediamine hydrochloride (20 mmol/L) and 0.5 mL ferric trichloride (30 mmol/L) were added to the reaction system, which was then incubated for 20 min at room temperature. After addition of l mL 10% trichloroacetic acid to precipitate albumin, the volume of the mixture was adjusted to 5 mL with distilled water and centrifuged for 5 min at 7400 *g*. The absorbance of the resulting supernatant solutions was read at 670 nm using a spectrophotometer, and H_2_S concentrations in the renal homogenates were calculated against the NaHS calibration curve. The proteins in the homogenates were quantified with the Coomassie brilliant blue colorimetric method (Jiancheng Biotechnology Research Institute, China). The results are reported as μmol H_2_S per mg protein.

### Assays of CSE and inflammatory factors in renal homogenates

CSE, TLR4, IL-10, IL-12, and TNF-α levels in the renal homogenates were determined using a rat enzyme-linked immunoadsorbent assay (ELISA) kit and commercially available antibodies (R&D Systems, USA). Results are reported as μmol per mg protein (CSE) and ng/mg protein (TLR4, IL-10, IL-12, and TNF-α).

### Statistical analysis

Data are reported as means±SD, and the statistical analyses were performed using the SPSS software version 16.0 (SPSS Inc., USA). Differences of the means observed in the experimental groups were analyzed by one-way analysis of variance (ANOVA) followed by Student-Newman-Keuls tests. Differences between two group means were analyzed with independent sample *t*-tests. P<0.05 was considered to be significant.

## Results

### H_2_S levels in renal tissue

**Figure 1:**
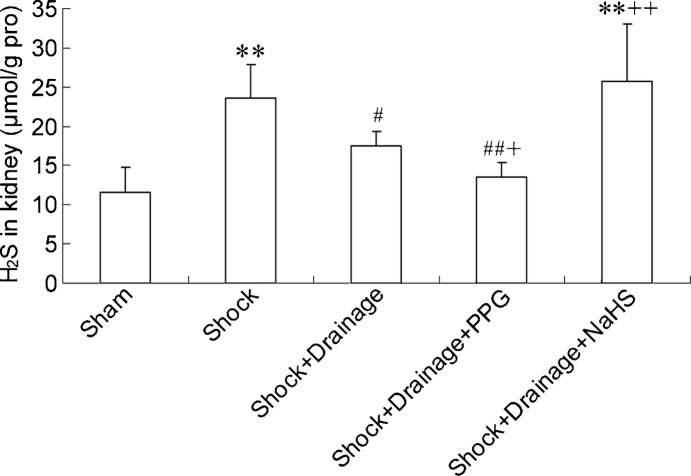
Changes in hydrogen sulfide (H_2_S) levels in rat kidneys. Data are reported as means±SD (µmol/g protein, n=6). PPG: D,L-propargylglycine; NaHS: sodium hydrosulfide hydrate. **P<0.01 *vs* the sham group; ^#^P<0.05, ^##^P<0.01 *vs* the shock group; ^+^P<0.05, ^++^P<0.01 *vs* the shock+drainage group (one-way ANOVA).

H_2_S levels in renal tissue from the shock group were significantly higher than those in the sham group (P<0.01, Figure 1). H_2_S levels in the shock+drainage group were significantly lower than those in the shock group (P<0.05). PPG administration significantly decreased H_2_S levels (P<0.05), which were significantly lower than those observed in the shock group (P<0.01). NaHS administration significantly increased H_2_S to levels similar to those in the shock group and significantly higher than those observed in the sham and shock+drainage groups (P<0.01).

### CSE levels in renal tissue

**Figure 2:**
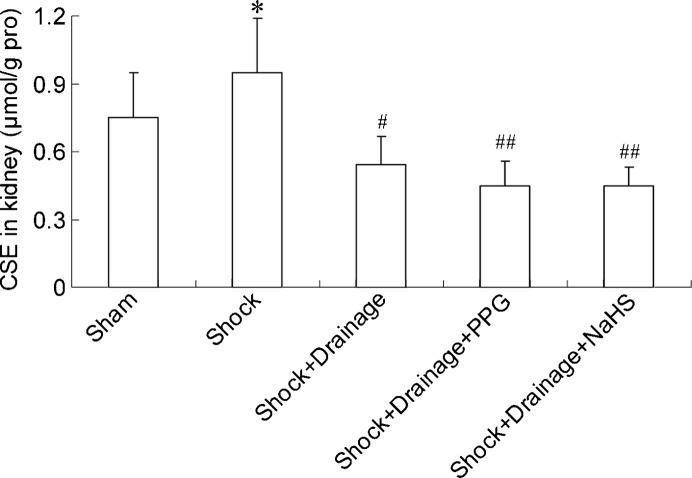
Changes in cystathionine-γ-lyase (CSE) levels in rat kidneys. Data are reported as means±SD (µmol/g protein, n=6). PPG: D,L-propargylglycine; NaHS: sodium hydrosulfide hydrate. *P<0.05 *vs* the sham group; ^#^P<0.05, ^##^P<0.01 *vs* the shock group (one-way ANOVA).

As shown in Figure 2, CSE levels in renal tissue from the shock group were significantly higher than those in tissues from the sham group (P<0.05). CSE levels in tissues from the shock+drainage, shock+drainage+PPG, and shock+drainage+NaHS groups were significantly lower than that of the shock group (P<0.05, P<0.01). The differences in CSE levels observed among the three experimental groups were not significant (P>0.05).

### Renal function indices

**Figure 3:**
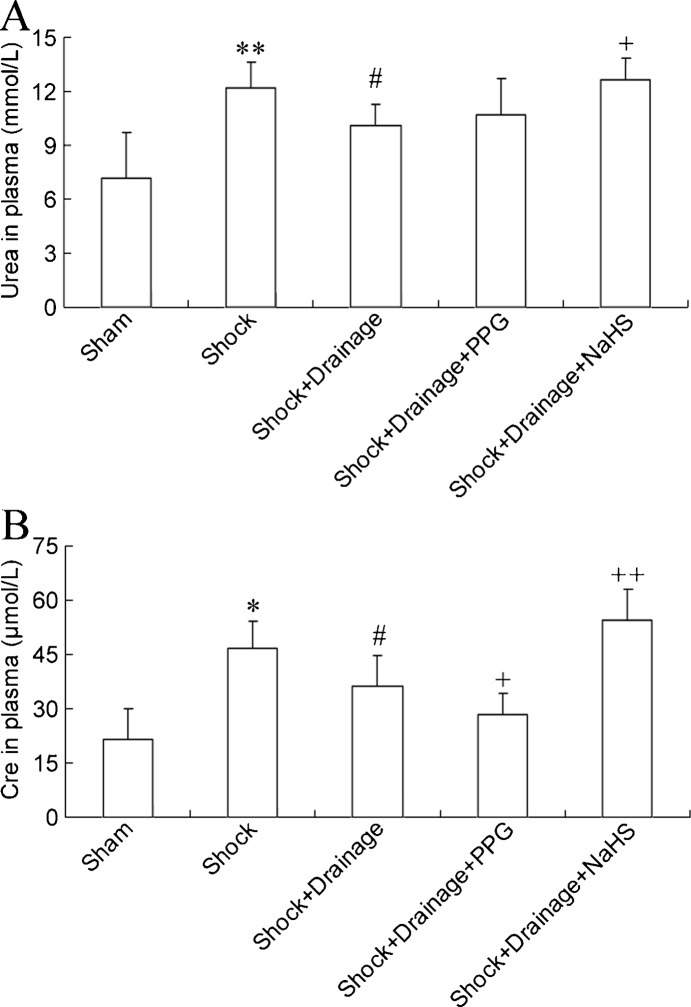
Changes in biochemical indices of renal function in rats. *A*, Urea in plasma; *B*, Creatinine (Cre) in plasma. Data are reported as means±SD (n=6). PPG: D,L-propargylglycine; NaHS: sodium hydrosulfide hydrate. *P<0.05, **P<0.01 *vs* the sham group; ^#^P<0.05 *vs* the shock group; ^+^P<0.05, ^++^P<0.01 *vs* the shock+drainage group (one-way ANOVA).

Figure 3 shows that hemorrhagic shock induced significant increases in plasma urea (P<0.05) and Cre (P<0.01) levels and that PHSML drainage decreased urea and Cre levels (P<0.05). PPG administration enhanced the effect of PHSML drainage on Cre (P<0.05), whereas NaHS administration reversed the effect of PHSML drainage on urea (P<0.05) and Cre (P<0.01) levels.

### Renal morphology

**Figure 4:**
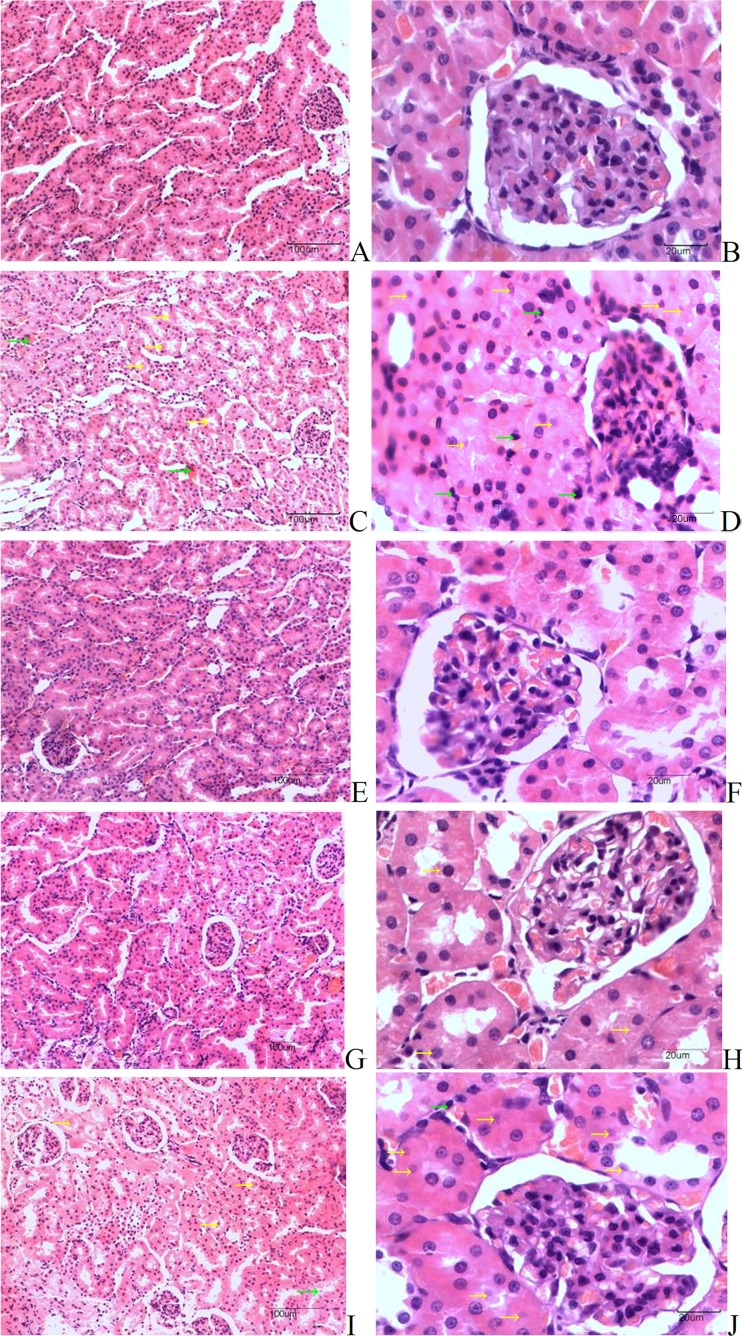
Changes in renal pathomorphology in rats (hematoxylin and eosin staining). *A*,*B*, sham group; *C*,*D*, shock group; *E*,*F*, shock+drainage group; *G*,*H*, shock+drainage+PPG group; *I*,*J*, shock+drainage+NaHS group. PPG: D,L-propargylglycine; NaHS: sodium hydrosulfide hydrate. Normal structure of renal glomeruli and tubules in the sham group is shown in *A* and *B*; protein casts (yellow arrows) and erythrocytes (green arrows) were found in renal tubule lumens of rats in the shock and shock+drainage+NaHS groups, as shown in *C*and *I*; tubular epithelial cells with necrosis (yellow arrows) and karyopyknosis (green arrows) were observed in the shock and shock+drainage+NaHS groups, as shown in *D* and *J*; nearly normal architecture of glomeruli and tubules in the shock+drainage and shock+drainage+PPG groups is shown in *E*, *F*, *G*, and *H*, plus mild edema of tubular epithelial cells (yellow arrows) in *H*.

The architecture of glomeruli and renal tubules of rats in the sham group appeared normal (Figure 4A and B). Protein casts and erythrocytes were occasionally found in renal tubule lumens of rats in the shock group along with tubular epithelial cells with necrosis, karyopyknosis, and acidophilic degeneration (Figure 4C and D). In the shock+drainage group, the glomeruli and tubules showed nearly normal architecture with normally arranged tubular epithelial cells containing integral nuclei with clear and complete membranes (Figure 4E and F). In the shock+drainage+PPG group, the glomeruli and tubules had nearly normal architecture with a few mildly edemic tubular epithelial cells (Figure 4G and H). Finally, in the shock+drainage+NaHS group, the injury to tubular epithelial cells was significantly greater than that seen in the shock+drainage group. Acidophilic degeneration and edema were also noted in the tubular epithelial cells in some regions of tissue (Figure 4I and J).

### TLR4 levels in renal tissue

**Figure 5:**
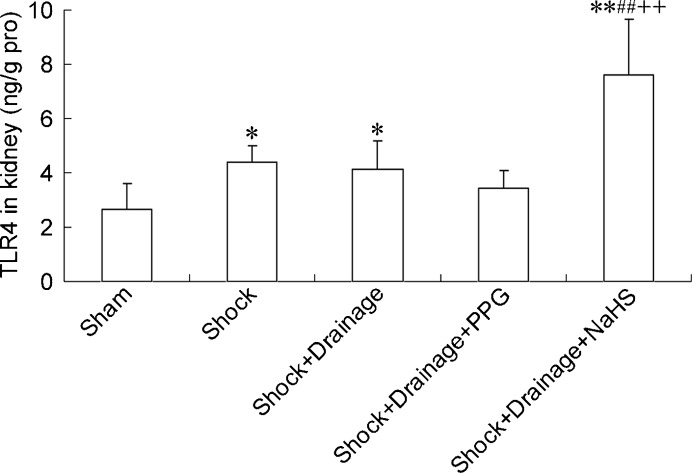
Changes in Toll-like receptor 4 (TLR4) levels in the renal homogenates of rats. Data are reported as means±SD (ng/g protein, n=6). PPG: D,L-propargylglycine; NaHS: sodium hydrosulfide hydrate. *P<0.05, **P<0.01 *vs* the sham group; ^##^P<0.01 *vs* the shock group; ^++^P<0.01 *vs* the shock+drainage group (one-way ANOVA).

TLR4 levels in renal homogenates of the shock and shock+drainage groups were significantly higher than levels in the sham group (P<0.05). TLR4 levels in renal homogenates of the shock+drainage+PPG group showed a decreasing trend, but no statistical difference was found compared with the shock group (P>0.05, Figure 5). However, NaHS administration significantly increased TLR4 levels compared with those in the sham, shock, and shock+drainage groups (P<0.01).

### IL-10 and IL-12 levels in renal tissue

**Figure 6:**
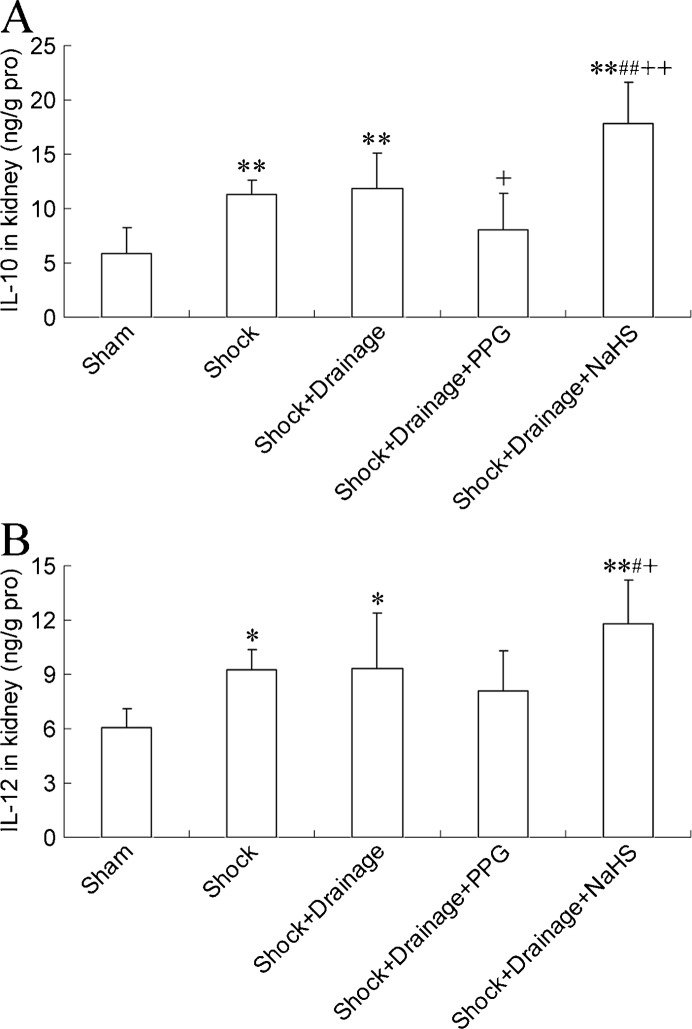
Changes in interleukin (IL)-10 (*A*) and IL-12 (*B*) levels in the renal homogenates of rats. Data are reported as means±SD (ng/g protein, n=6). PPG: D,L-propargylglycine; NaHS: sodium hydrosulfide hydrate. *P<0.05, **P<0.01 *vs* the sham group; ^#^P<0.05, ^##^P<0.01 *vs*the shock group; ^+^P<0.05, ^++^P<0.01 *vs* the shock+drainage group (one-way ANOVA).

IL-10 and IL-12 levels in renal homogenates from the shock and shock+drainage groups were significantly higher than those in the sham group (P<0.05, Figure 6). PPG administration significantly decreased IL-10 levels (P<0.05), but the decrease in IL-12 levels was not significant (P>0.05). NaHS administration significantly enhanced IL-10 (P<0.01) and IL-12 (P<0.05) levels compared to the shock+drainage group, as well as with significant differences compared to the sham and shock groups (P<0.05, P<0.01).

### TNF-α levels in renal tissue

**Figure 7:**
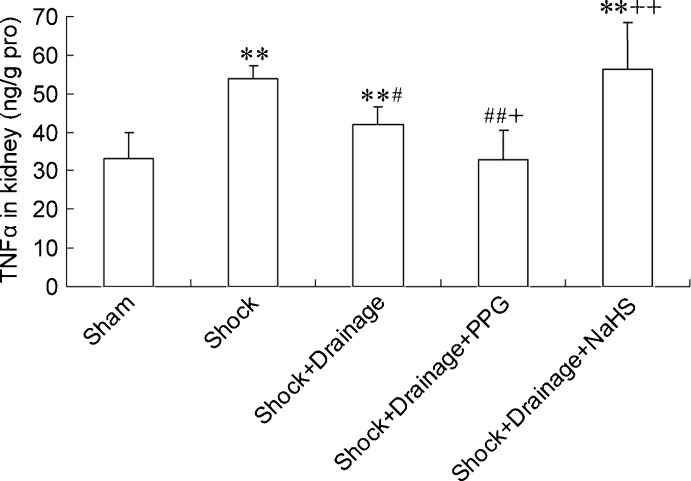
Changes in tumor necrosis factor (TNF)-α levels in the renal homogenates of rats. Data are reported as means±SD (ng/g protein, n=6). PPG: D,L-propargylglycine; NaHS: sodium hydrosulfide hydrate. **P<0.01 *vs* the sham group; ^#^P<0.05, ^##^P<0.01 *vs*the shock group; ^+^P<0.05, ^++^P<0.01 *vs* the shock+drainage group (one-way ANOVA).

TNF-α levels in renal homogenates from the shock and shock+drainage groups were significantly higher than those in the sham group (P<0.01), and the index in the shock+drainage group was lower than that in the shock group (P<0.05, Figure 7). PPG administration significantly decreased TNF-α levels, with significant differences in the levels measured in the shock and shock+drainage groups (P<0.05 and P<0.01, respectively). NaHS administration significantly increased TNF-α levels in renal homogenates (P<0.01).

## Discussion

In the present study, we assayed H_2_S levels in renal tissues to investigate the role of H_2_S in PHSML drainage responsible for alleviation of AKI in hemorrhagic-shocked rats. We found that the mean H_2_S level was significantly increased in the shock group compared with the sham group. H_2_S level was decreased by PHSML drainage and further reduced by PPG administration, but this effect was reversed by NaHS administration. Further investigation revealed that CSE levels were significantly increased in renal tissues from the shock group. Thus, PHSML drainage and PPG decreased CSE levels in renal tissues from the shock group, but NaHS had no significant effect on CSE levels, indicating that the decrease in H_2_S was achieved by inhibition of CSE activity by PHSML drainage. NaHS, under the influence of CSE, directly generates H_2_S, which indicates that exogenous NaHS had no effect on CSE activity.

In addition to the effect of PHSML drainage on AKI resulting from inhibition of CSE activity and decreased H_2_S levels, we found that plasma urea and Cre, which are biochemical indicators of renal function, significantly increased in the shock group and decreased after PHSML drainage. PPG enhanced the role of PHSML drainage, but NaHS had the opposite effect, indicating that NaHS reversed the impact of PHSML drainage, which is consistent with the observed differences in renal tissue morphology. Furthermore, H_2_S reduced the effect of PHSML drainage and aggravated structural damage to the kidneys of hemorrhagic-shocked rats.

In an animal model of hemorrhagic shock (17), H_2_S promoted the systemic inflammatory response syndrome and myeloperoxidase activity in lung tissue. In addition, PPG decreased plasma TNF-α, IL-1, IL-6, and IL-10 levels, suggesting that H_2_S plays an important role in organ injury following hemorrhagic shock. H_2_S was also involved in inflammatory responses to severe burns in mice and renal ischemia-reperfusion in rats (18,19). We found that PHSML drainage reduced TNF-α levels in shocked rats and that this effect was strengthened by PPG. However, NaHS administration significantly increased TNF-α levels. H_2_S reduction was also related to the mechanism of PHSML drainage for reducing renal injury and the inflammatory response - processes in which H_2_S plays a negative role.

TLR4 is an important receptor of the innate immune system that recognizes the specific molecular structure of highly conserved binding sites of certain pathogens or their products. TLR4 initiates an inflammatory response through the identification and binding of different pathogen-associated molecular species and is involved in uncontrolled inflammatory responses following hemorrhagic shock (20,21). TLR4 is also involved in lipopolysaccharide-induced kidney injury (22). Our results showed that TLR4, IL-10, and IL-12 levels were significantly increased in the renal tissues of rats in shock, suggesting that TLR4 mediated renal injury after hemorrhagic shock. However, PHSML drainage had no effect on these markers, suggesting that the role of PHSML drainage in alleviating AKI was unrelated to TLR4 or that it resulted from the short observation time in this study. Thus, observation time should be extended in future studies to further clarify the role of TLR4 in AKI induced by PHSML return. Moreover, NaHS, an H_2_S donor, increased TLR4, IL-10, and IL-12 levels in the renal tissues of rats in the shock+drainage group, but PPG reduced the inflammatory response. Therefore, we can conclude that H_2_S induces TLR4 to aggravate the renal tissue inflammatory response.

Pretreatment with PPG decreased plasma urea levels and H_2_S levels in the kidneys of hemorrhagic-shocked rats as reported by Mok et al. (13), who evaluated the role of H_2_S in AKI following hemorrhagic shock. Our aim, however, was to focus on the relationship between H_2_S and PHSML drainage, so we did not study the effect of PPG or NaHS on AKI, which is a limitation of this study. The roles of PHSML drainage and PPG on AKI induced by hemorrhagic shock should be compared in future studies.

In summary, PHSML drainage had beneficial effects on AKI and renal dysfunction following hemorrhagic shock. AKI amelioration was associated with decreased H_2_S levels and the downregulation of H_2_S-mediated inflammation. These findings provide a sound experimental basis for the clinical prevention and treatment of AKI in critically ill patients with a focus on the lymphatic pathway and H_2_S regulation.
